# Integrating Urinary Sodium into the Larissa Heart Failure Risk Score Improves Early Risk Stratification in Acute Heart Failure

**DOI:** 10.3390/jcdd13090405

**Published:** 2026-08-23

**Authors:** Nikolaos Chrysakis, Dimitrios E. Magouliotis, Ioannis Leventis, Evangelia Katsimperi, Grigorios Giamouzis, Filippos Triposkiadis, John Skoularigis, Andrew Xanthopoulos

**Affiliations:** 1 Department of Cardiology, University Hospital of Larissa, Faculty of Medicine, University of Thessaly, 41100 Larissa, Greece; nchrysakis@uth.gr (N.C.); iolevent@med.uth.gr (I.L.); euakats96@gmail.com (E.K.); grgiamouzis@uth.gr (G.G.); ftriposk@med.uth.gr (F.T.); iskoular@uth.gr (J.S.); 2 Department of Cardiac Surgery Research, Lankenau Institute for Medical Research, Wynnewood, PA 19096, USA; magouliotisd@mlhs.org; 3 School of Medicine, European University Cyprus, P.O. Box 22006, Nicosia 2404, Cyprus

**Keywords:** heart failure, urinary sodium, diuretic resistance, prognosis, decompensation

## Abstract

(1) Introduction: Early identification of patients at high risk of recurrent events after hospitalization for acute decompensated heart failure (ADHF) remains challenging. The Larissa Heart Failure Risk Score (LHFRS) is a simple prognostic tool based on hypertension, coronary artery disease, and red blood cell distribution width. Urinary sodium has recently emerged as an objective marker of natriuretic response and decongestion. We prospectively evaluated whether incorporation of urinary sodium improves the prognostic performance of the LHFRS. (2) Methods: This prospective single-center observational study enrolled 130 consecutive adults hospitalized with ADHF. Clinical, laboratory, electrocardiographic, and echocardiographic data were collected at admission. Spot urinary sodium and chloride were measured at admission and 2 h after intravenous loop diuretic administration according to a standardized decongestion protocol. The primary endpoint was heart failure rehospitalization within 3 months. Secondary endpoints included all-cause mortality and the composite of death or heart failure rehospitalization. Multivariable logistic regression with bootstrap internal validation (1000 resamples) was used to identify independent predictors of outcomes. (3) Results: The study population included patients across the spectrum of heart failure phenotypes (HFrEF 58%, HFmrEF 7%, HFpEF 35%). During follow-up, 48 patients (36.9%) experienced heart failure rehospitalization and 23 (17.7%) died. The LHFRS independently predicted 3-month rehospitalization (B = 0.490, *p* = 0.041). Admission urinary sodium and 2-h urinary sodium provided incremental prognostic information beyond the LHFRS and remained independently associated with rehospitalization after multivariable adjustment (*p* = 0.008 and *p* = 0.001, respectively). Urinary chloride demonstrated similar prognostic associations, whereas conventional renal biomarkers, including serum creatinine, urea, estimated glomerular filtration rate, serum sodium, and NT-proBNP, did not consistently retain independent prognostic significance. The LHFRS was also significantly associated with the composite endpoint of death or rehospitalization (B = 1.173, *p* = 0.002), while its association with mortality alone was not statistically significant (B = −5.551, *p* = 0.256). (4) Conclusions: Lower admission and 2-h urinary sodium concentrations were associated with 3-month HF rehospitalization after adjustment for the LHFRS. These findings are hypothesis-generating and require confirmation in larger, externally validated multicenter cohorts.

## 1. Introduction

Acute decompensated heart failure (ADHF) remains one of the leading causes of hospitalization among older adults and is associated with substantial morbidity, mortality, and healthcare expenditure worldwide. Despite major advances in guideline-directed medical therapy, patients remain particularly vulnerable during the first months following hospital discharge, when the risk of recurrent decompensation, rehospitalization, and death is highest. Early identification of patients at increased risk of adverse outcomes therefore represents a major clinical priority, allowing closer surveillance, optimization of medical therapy, and timely implementation of disease-management strategies [1,2].

Several prognostic models have been developed to improve risk stratification in patients with heart failure. However, many incorporate numerous clinical, laboratory, and imaging variables, limiting their practicality in routine clinical practice. The Larissa Heart Failure Risk Score (LHFRS) was developed as a simple bedside prognostic tool based on only three readily available variables: history of hypertension, history of coronary artery disease, and red blood cell distribution width (RDW) [3,4,5,6]. Despite its simplicity, the LHFRS has demonstrated good prognostic performance for predicting adverse outcomes following hospitalization for acute heart failure and has subsequently been externally evaluated in independent patient populations [5,6]. Nevertheless, the score is based exclusively on static baseline characteristics and does not incorporate dynamic markers reflecting the patient’s response to decongestive therapy [7].

Congestion is the predominant pathophysiological abnormality in ADHF and the principal cause of hospitalization [2,8,9]. Contemporary evidence suggests that persistent congestion at hospital discharge is among the strongest predictors of early rehospitalization and mortality. Consequently, recent therapeutic strategies have shifted from symptom-guided treatment toward objective assessment of decongestion. Among the available biomarkers, spot urinary sodium has emerged as a simple, inexpensive, and physiologically meaningful indicator of natriuretic response to loop diuretics [10]. Low urinary sodium concentrations identify patients with impaired renal sodium handling, inadequate diuretic responsiveness, and persistent congestion, all of which are associated with worse clinical outcomes [11]. These observations have led to the incorporation of urinary sodium into the European Society of Cardiology decongestion algorithm for patients hospitalized with acute heart failure [2].

Although urinary sodium has consistently demonstrated prognostic value in acute heart failure, its incremental contribution when combined with established clinical risk scores has not been adequately investigated. Integrating a dynamic marker of renal response with a simple clinical risk model may improve early risk stratification by capturing complementary aspects of heart failure pathophysiology, including cardiovascular reserve, renal perfusion, tubular sodium handling, and responsiveness to diuretic therapy.

Therefore, the present prospective study evaluated whether incorporation of urinary sodium into the Larissa Heart Failure Risk Score enhances prediction of short-term clinical outcomes in patients hospitalized with ADHF. Specifically, we investigated whether admission and early post-diuretic urinary sodium measurements provide incremental prognostic information beyond the LHFRS for predicting 3-month heart failure rehospitalization, all-cause mortality, and the composite of death or rehospitalization.

## 2. Materials and Methods

### 2.1. Study Design

This was a prospective, single-center observational study conducted at the University General Hospital of Larissa, Greece. Consecutive adult patients (≥18 years) hospitalized with ADHF between 15 December 2023 and 15 December 2024 were prospectively enrolled. The diagnosis of ADHF was established according to contemporary European Society of Cardiology criteria [1,2]. Patients were excluded if they presented with acute coronary syndrome, prior heart transplantation, left ventricular assist device support, surgically corrected congenital heart disease, chronic hemodialysis, sepsis, or blood-product transfusion during the index hospitalization.

### 2.2. Clinical Assessment

Baseline demographic characteristics, medical history, physical examination findings, laboratory measurements, electrocardiographic data, and transthoracic echocardiographic parameters were obtained at hospital admission (Supplementary Material). Loop diuretic therapy was administered according to the contemporary ESC decongestion algorithm. Patients not receiving chronic loop diuretics received intravenous furosemide 20–40 mg, whereas patients receiving chronic oral therapy received an intravenous dose equivalent to or up to twice their previous daily oral dose. Spot urinary sodium and chloride concentrations were measured immediately before diuretic administration and again 2 h after treatment. Urine output was recorded during the first 6 h following treatment [2,10]. Three months after discharge, telephone follow-up was performed to document survival status, death, and any symptoms or signs of decompensated heart failure leading to medical evaluation.

### 2.3. Larissa Heart Failure Risk Score

The Larissa Heart Failure Risk Score (LHFRS) is a simple score derived from three factors (history of hypertension [yes = 0, no = 2]; history of coronary artery disease/myocardial infarction [yes = 1, no = 0]; and red blood cell distribution width [RDW] ≥ 15% [yes = 1, no = 0]; best = 0, worst = 4) [6].

### 2.4. Endpoints

The primary endpoint was heart failure rehospitalization within 3 months following discharge. Secondary endpoints included all-cause mortality within the first 3 months after discharge and the composite endpoint of death or rehospitalization during the same period.

### 2.5. Statistical Analysis

Continuous variables are presented as mean ± SD when approximately normally distributed and as median (IQR) when non-normally distributed or conventionally skewed (e.g., NT-proBNP, urine electrolytes, urine volume). Categorical variables are presented as n (%). Associations between clinical variables and outcomes were evaluated using multivariable logistic regression. To account for the relatively modest sample size, internal validation was performed using non-parametric bootstrap resampling (1000 iterations). A two-sided *p* < 0.05 was considered statistically significant. All analyses were conducted using SPSS (Version 25, Windows, IBM Corp., Armonk, NY, USA). Missing values were not imputed. Available N indicates the number of real, non-missing observations used for each calculation.

### 2.6. Ethical Approval

The study complied with the principles of the Declaration of Helsinki. It received approval from the Ethics and Deontology Committee and the Scientific Council of the hospital, as well as from the Assembly of the Department of Medicine, School of Health Sciences, University of Thessaly. Written informed consent was obtained from every patient for the handling of personal data after full explanation of the purpose, procedures, and nature of the intervention. For patients unable to provide consent, consent was obtained from the responsible accompanying person.

## 3. Results

### 3.1. Study Population

**Figure 1:**
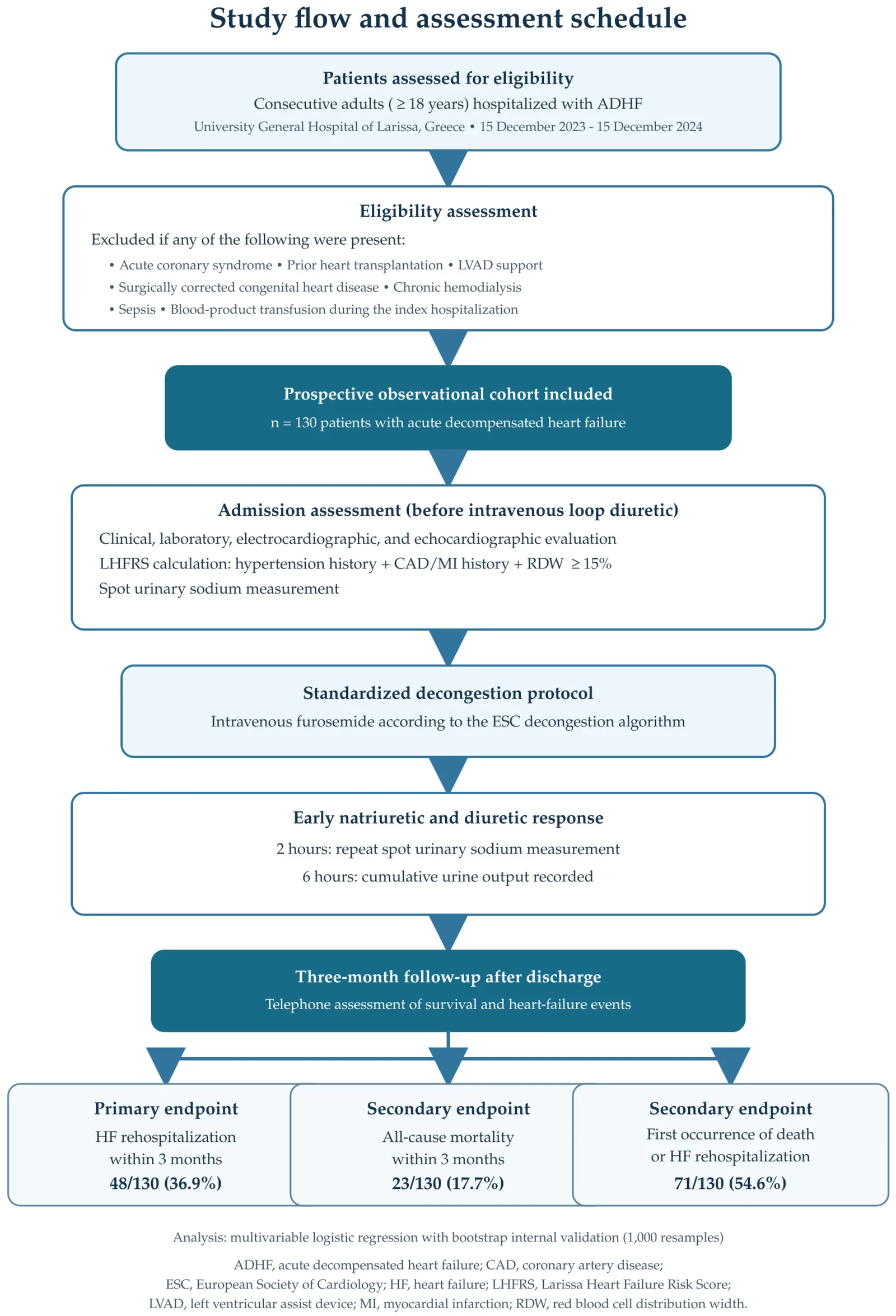
Study flowchart.

A total of 130 patients with ADHF were included (Figure 1). The mean age was 78.8 ± 10.8 years, and 58.5% were male. Heart failure phenotypes included HFrEF (58.5%), HFpEF (34.6%), and HFmrEF (6.9%), while approximately one-quarter of patients had de novo heart failure. Hypertension (71.5%), atrial fibrillation (52%), and coronary artery disease (35.4%) were the most prevalent comorbidities. Median admission urinary sodium was 55 mmol/L, increasing to 102 mmol/L two hours following intravenous diuretic administration (Table 1).

### 3.2. Clinical Outcomes

During the study follow-up, 48 patients (36.9%) of the overall cohort were rehospitalized, and 23 patients (17.7%) died (Table 2).

### 3.3. Predictors of Rehospitalization

The LHFRS remained independently associated with 3-month rehospitalization (B = 0.490, *p* = 0.041). Among renal biomarkers, admission urinary sodium and 2-h urinary sodium demonstrated the strongest independent associations with rehospitalization (*p* = 0.008 and *p* = 0.001, respectively). Admission and post-diuretic urinary chloride also remained significant predictors. In contrast, serum creatinine, urea, eGFR, and NT-proBNP showed weaker or non-significant associations after multivariable adjustment (Table 3).

### 3.4. Predictors of the Composite Endpoint

For the composite endpoint of death or rehospitalization, the LHFRS remained independently associated with adverse outcome (B = 1.173, *p* = 0.002). Markers of renal function, natriuretic peptides, urinary sodium, urinary chloride and urine output were also significantly associated with the composite endpoint (Table 4).

### 3.5. Predictors of Mortality

For all-cause mortality, the LHFRS was not independently associated with outcome.

Instead, serum sodium, urinary creatinine, and urine volume were the strongest independent predictors (Table 5).

## 4. Discussion

This prospective study demonstrates that combining the Larissa Heart Failure Risk Score (LHFRS) with urinary sodium measurements improves early risk stratification in patients hospitalized with ADHF. Three principal findings emerge.

First, the LHFRS retained independent prognostic value for predicting heart failure rehospitalization within three months, confirming the robustness of this simple bedside risk score in a contemporary cohort of patients with acute heart failure. Second, both admission and early post-diuretic urinary sodium concentrations provided incremental prognostic information beyond the LHFRS, supporting the concept that dynamic assessment of natriuretic response complements traditional clinical risk factors. Third, urinary sodium outperformed several conventional markers of renal function and congestion, suggesting that direct assessment of renal sodium handling may more accurately reflect the pathophysiological mechanisms underlying recurrent decompensation.

The present study confirms the prognostic utility of the LHFRS in patients hospitalized with acute heart failure. Although the score is intentionally simple, relying on only hypertension, coronary artery disease, and RDW, these variables capture distinct biological pathways involved in disease progression [12]. The absence of hypertension likely reflects impaired cardiovascular reserve and reduced capacity to maintain systemic perfusion during decompensation [13]. Coronary artery disease identifies patients with irreversible myocardial injury and fibrosis, increasing susceptibility to recurrent congestion and ventricular dysfunction [14]. Elevated RDW has consistently been associated with inflammation, iron dysregulation, nutritional abnormalities, and impaired erythropoiesis, all of which characterize advanced heart failure [12]. Importantly, the present study confirms that these readily available variables continue to provide meaningful prognostic information despite the increasing complexity of contemporary heart failure populations and widespread implementation of guideline-directed medical therapy.

The principal novelty of this study is the demonstration that urinary sodium provides incremental prognostic information beyond an established clinical risk score. Unlike serum creatinine or estimated glomerular filtration rate, urinary sodium directly reflects the kidney’s ability to excrete sodium in response to congestion and loop diuretics [10]. Reduced urinary sodium indicates persistent neurohormonal activation, impaired renal perfusion, increased tubular sodium reabsorption, and early diuretic resistance [15]. Consequently, urinary sodium captures an aspect of heart failure biology that conventional renal biomarkers cannot adequately assess. Serum creatinine primarily reflects glomerular filtration, whereas urinary sodium reflects the integrated physiological response involving renal perfusion, tubular function, neurohormonal activation, and pharmacological responsiveness.

The observation that both admission and 2-h urinary sodium independently predicted rehospitalization further supports the concept that early natriuretic response represents a dynamic biomarker of decongestion. Patients unable to achieve adequate sodium excretion despite diuretic therapy probably remain congested despite apparent clinical improvement, predisposing them to recurrent hospitalization during the vulnerable post-discharge period.

Our findings are consistent with a growing body of evidence demonstrating the prognostic importance of urinary sodium in acute heart failure. Several observational studies have shown that low spot urinary sodium identifies patients with poor diuretic response, prolonged hospitalization, and increased mortality [16]. More recently, studies including DIURESIS-AHF [17], ROSE-AHF [18], ENACT-HF [15], and PUSH-AHF [10], DECONGEST [19] have highlighted the clinical utility of natriuresis-guided decongestion strategies, while the ADVOR trial emphasized the importance of achieving complete decongestion during hospitalization. The present study extends these observations in two important ways. First, rather than evaluating urinary sodium as an isolated biomarker, we demonstrate its incremental value when combined with an established clinical prognostic model. Second, our findings suggest that integrating static clinical predictors with dynamic markers of renal response may provide a more comprehensive assessment of patient risk than either approach alone. This integrated approach is biologically plausible because recurrent heart failure reflects both baseline disease severity and the effectiveness of decongestion during the index hospitalization [20].

### 4.1. Clinical Implications

These findings have several practical implications. Because urinary sodium is inexpensive, rapidly available, and already incorporated into contemporary decongestion algorithms, its routine measurement requires little additional infrastructure. Combining urinary sodium with the LHFRS could facilitate identification of patients requiring intensified diuretic therapy, prolonged hospitalization until adequate decongestion is achieved, earlier outpatient reassessment after discharge, or enrollment in multidisciplinary heart failure management programs. Importantly, this strategy aligns with the current movement toward individualized treatment guided by objective markers of congestion rather than symptoms alone [21]. Future studies should evaluate whether urinary sodium-guided risk stratification can improve clinical outcomes by directing more intensive post-discharge management.

### 4.2. Study Limitations

Several limitations should be acknowledged. First, this was a prospective single-center study with a relatively modest sample size and incomplete availability of some decongestion variables (i.e., individual cumulative intravenous diuretic doses or body weight changes), which limited statistical power and generalizability. Second, although bootstrap resampling demonstrated internal robustness of the findings, external validation in independent cohorts is required before widespread clinical implementation. Third, the number of deaths during follow-up was small, and the extreme coefficient magnitudes may reflect sparse-data instability, multicollinearity, or model overfitting. Accordingly, the mortality findings should be considered hypothesis-generating, and overfitting cannot be excluded. Finally, urinary sodium was measured according to a standardized protocol at a single institution, and reproducibility across different healthcare systems should be confirmed.

## 5. Conclusions

In patients hospitalized with acute decompensated heart failure, combining the Larissa Heart Failure Risk Score with early urinary sodium assessment significantly improves short-term risk stratification. Urinary sodium provides prognostic information beyond conventional clinical and renal biomarkers by capturing the dynamic renal response to congestion and diuretic therapy. This combined approach represents a practical and biologically grounded strategy for identifying patients at increased risk of early recurrent decompensation and may facilitate more personalized in-hospital management and post-discharge follow-up. External multicenter validation is warranted before routine clinical implementation.

## Figures and Tables

**Table 1:** Baseline characteristics and laboratory findings of the study population.

Demographic characteristics	Age, years	78.8 ± 10.8 (Ν = 130)
	Male sex	76 (58.5%) (Ν = 130)
	Female sex	54 (41.5%) (Ν = 130)
Heart failure phenotype and risk score	HF phenotype: HFrEF	76 (58.5%) (Ν = 130)
	HF phenotype: HFmrEF	9 (6.9%) (Ν = 130)
	HF phenotype: HFpEF	45 (34.6%) (Ν = 130)
	De novo heart failure	32 (24.6%) (Ν = 130)
	Larissa Heart Failure Risk Score	1.0 (1.0–2.0) (Ν = 130)
	Larissa Heart Failure Risk Score (MI)	1.0 (1.0–2.0) (Ν = 130)
	NYHA class: 3	74 (56.9%) (Ν = 130)
	NYHA class: 2	35 (26.9%) (Ν = 130)
	NYHA class: 4	19 (14.6%) (Ν = 130)
	NYHA class: 1	2 (1.5%) (Ν = 130)
Medical history and comorbidities	History of arterial hypertension	93 (71.5%) (Ν = 130)
	History of coronary artery disease	46 (35.4%) (Ν = 130)
	History of acute coronary syndrome	23 (17.7%) (Ν = 130)
	Home cardiovascular/medical treatment recorded	123 (95.3%) (Ν = 129)
	Comorbidities recorded	122 (97.6%) (Ν = 125)
Clinical examination at admission	Respiratory auscultation: rales	82 (61.5%) (Ν = 130)
	Respiratory auscultation: normal	23 (17,7%) (Ν = 130)
	Peripheral oedema	84 (65.1%) (Ν = 130)
	Jugular venous distension	70 (53.8%) (Ν = 130)
Arterial blood gases at admission	pH	7.4 (7.4–7.5) (Ν = 74)
	PaO_2_, mmHg	47.0 ± 23.7 (Ν = 74)
	PaCO_2_, mmHg	42.5 (34.2–47.8) (Ν = 74)
	HCO_3_^−^, mmol/L	26.9 ± 6.0 (Ν = 74)
Blood laboratory findings at admission	Haematocrit, %	37.9 ± 5.4 (Ν = 130)
	Haemoglobin, g/dL	12.2 ± 1.9 (Ν = 130)
	RDW, %	16.0 (14.8–18.0) (Ν = 130)
	Serum urea, mg/dL	57.0 (42.2–79.8) (Ν = 130)
	Serum creatinine, mg/dL	1.2 (1.0–1.6) (Ν = 127)
	Urea/creatinine ratio	49.0 (38.9–57.6) (Ν = 128)
	eGFR-CKD-EPI, mL/min/1.73 m^2^	52.8 (36.2–71.6) (Ν = 128)
	FeNa at admission, %	0.9 (0.3–2.3) (Ν = 114)
	NT-proBNP at admission, ng/L	5305.0 (2940.0–9997.5) (Ν = 106)
	Serum sodium, mmol/L	139.0 (135.5–141.0) (Ν = 128)
Urinary electrolytes and urine output Urinary electrolytes and urine output	Spot urine sodium at admission, mmol/L	55.2 (31.9–90.4) (Ν = 130)
	Spot urine sodium at 2 h, mmol/L	102.2 (76.8–121.0) (Ν = 127)
	Spot urine chloride at admission, mmol/L	49.1 (25.6–87.7) (Ν = 125)
	Spot urine chloride at 2 h, mmol/L	98.9 (75.3–117.0) (Ν = 121)
	Spot urine creatinine at admission, mg/dL	57.2 (27.7–93.3) (Ν = 121)
	Spot urine creatinine at 2 h, mg/dL	11.8 (7.0–21.3) (Ν = 118)
	Urine volume at 6 h, mL	1600.0 (1000.0–2500.0) (Ν = 87)
	Urine volume at 24 h, mL	3000.0 (2400.0–4300.0) (Ν = 51)
Echocardiographic findings	LVEF, %	38.5 ± 13.8 (Ν = 130)
	LVEDD, mm	55.0 (55.0–55.0) (Ν = 130)
	LV filling pressure E/e′ lateral	11.0 (10.0–13.0) (Ν = 127)
	Left atrial dimension, mm	40.0 (40.0–42.0) (Ν = 129)
	RV basal diameter, mm	40.0 (40.0–40.0) (Ν = 130)
	TDI RV S′, cm/s	10.0 (10.0–10.0) (Ν = 129)
	RVSP, mmHg	50.0 (40.0–60.0) (Ν = 83)
	IVC diameter, mm	22.0 (20.0–24.0) (Ν = 130)
	LV wall thickness/pattern: EΦO	95 (73.1%) (Ν = 130)
	LV wall thickness/pattern: CLVH	29 (22.3%) (Ν = 130)
	LV wall thickness/pattern: ELVH	6 (6.6%) (Ν = 130)
	Mitral valve regurgitation/prosthesis field: 2+/4+	52 (40.3%) (Ν = 130)
	Mitral valve regurgitation/prosthesis field: 1+/4+	28 (21.7%) (Ν = 130)
	Mitral valve regurgitation/prosthesis field: 1–2+/4+	19 (14.7%) (Ν = 130)
	Mitral valve regurgitation/prosthesis field: 3+/4+	15 (11.6%) (Ν = 130)
	Mitral valve regurgitation/prosthesis field: 2–3+/4+	7 (5.4%) (Ν = 130)
	Mitral valve regurgitation/prosthesis field: 3–4+/4+	4 (3.2%) (Ν = 130)
	Respiratory variation of IVC: <50%	74 (57%) (Ν = 130)
	Respiratory variation of IVC: >50%	56 (43.1%) (Ν = 130)
Electrocardiographic findings	ECG rhythm: AF	68 (52.3%) (Ν = 130)
	ECG rhythm: SR	53 (40.9%) (Ν = 130)
	ECG rhythm: PACED	10 (7.7%) (Ν = 130)
	ECG ischemic changes	18 (13.8%) (Ν = 130)

Abbreviations: AF, atrial fibrillation; eGFR CKD-EPI, estimated Glomerular Filtration Rate Chronic Kidney Disease Epidemiology Collaboration equation; ECG, electrocardiogram; FeNa, fractional excretion of sodium; HF, heart failure; HFmrEF, heart failure with mildly reduced ejection fraction; HFpEF, heart failure with preserved ejection fraction; HFrEF, heart failure with reduced ejection fraction; IQR, interquartile range; IVC, inferior vena cava; LVEDD, left ventricular end-diastolic diameter; LVEF, left ventricular ejection fraction; NT-proBNP, N-terminal pro-B-type natriuretic peptide; NYHA, New York Heart Association; RVSP, right ventricular systolic pressure; SD, standard deviation.

**Table 2:** Clinical outcomes during follow-up.

Outcomes	Available N	Overall Cohort
Rehospitalization within 3 months	130	48 (36.9%) (Ν = 130)
Death within 3 months	130	23 (17.7%) (Ν = 130)
Death and/or rehospitalization *	130	71 (54.6%) (Ν = 130)

* For the composite endpoint, only the first qualifying event was counted. Clinical outcomes are presented as N (%). No artificial values or imputation were used.

**Table 3:** Logistic regression for the primary outcome.

Variables	B	*p* Value
Larissa Heart Failure Risk Score	0.490	0.041
Urea serum (mg/dL)	0.016	0.063
Creatinine serum (mg/dL)	0.662	0.070
eGFR-CKD-EPI	−0.037	0.057
NT-proBNP at admission (ng/L)	0.000	0.069
Sodium serum (mmol/L)	−0.088	0.052
Spot sodium urine at admission (mmol/L)	−0.081	0.008
Spot sodium urine at 2 h (mmol/L)	−0.186	0.001
Spot chloride urine at admission (mmol/L)	−0.082	0.008
Spot chloride urine at 2 h (mmol/L)	−0.189	0.004
Spot creatinine urine at admission (mg/dL)	−0.004	0.076
Spot creatinine urine at 2 h (mg/dL)	−0.021	0.076
Urine volume at 6 h (ml)	0.000	0.066
Urine volume at 24 h (ml)	0.000	0.092

Abbreviations: Same as Table 1.

**Table 4:** Logistic regression for the outcome of Death and/or Rehospitalization.

Variables	B	*p* Value
Larissa Heart Failure Risk Score	1.173	0.002
Urea serum (mg/dL)	0.142	0.002
Creatinine serum (mg/dL)	0.836	0.005
eGFR-CKD-EPI	−0.036	0.003
NT-proBNP at admission (ng/L)	0.000	0.005
Sodium serum (mmol/L)	−0.847	0.002
Spot sodium urine at admission (mmol/L)	−0.272	0.002
Spot sodium urine at 2 h (mmol/L)	−0.520	0.002
Spot chloride urine at admission (mmol/L)	−0.222	0.002
Spot chloride urine at 2 h (mmol/L)	−0.452	0.002
Spot creatinine urine at admission (mg/dL)	−0.048	0.002
Spot creatinine urine at 2 h (mg/dL)	−0.485	0.002
Urine volume at 6 h (ml)	−0.004	0.002
Urine volume at 24 h (ml)	−0.001	0.002

Abbreviations: Same as Table 1.

**Table 5:** Logistic regression for the outcome of death *.

Variables	B	*p* Value
Larissa Heart Failure Risk Score	−5.551	0.256
Urea serum (mg/dL)	0.232	0.472
Creatinine serum (mg/dL)	−130.956	0.067
eGFR-CKD-EPI	−1.033	0.075
NT-proBNP at admission (ng/L)	0.001	0.276
Sodium serum (mmol/L)	−4.024	0.008
Spot sodium urine at admission (mmol/L)	−0.260	0.512
Spot sodium urine at 2 h (mmol/L)	−0.046	0.917
Spot chloride urine at admission (mmol/L)	−1.658	0.087
Spot chloride urine at 2 h (mmol/L)	−1.900	0.154
Spot creatinine urine at admission (mg/dL)	−0.531	0.004
Spot creatinine urine at 2 h (mg/dL)	−3.459	0.004
Urine volume at 6 h (mL)	−0.041	0.031
Urine volume at 24 h (mL)	−0.010	0.063

* The mortality model is exploratory and hypothesis-generating due to the small number of events. Abbreviations: Same as Table 1.

## Data Availability

Data is contained within the article or Supplementary Material.

## Supplementary Materials

The following supporting information can be downloaded at: https://www.mdpi.com/article/10.3390/jcdd13090405/s1, Variables Recorded at Admission.
