# Supplementary material for: Integrating Urinary Sodium into the Larissa Heart Failure Risk Score Improves Early Risk Stratification in Acute Heart Failure
Source: J Cardiovasc Dev Dis. 2026 Aug 23;13(9):405. doi: 10.3390/jcdd13090405 (PMC13607949; doi:10.3390/jcdd13090405)
Supplement: Supplementary file 1 [file jcdd-13-00405-s001.zip › jcdd-4502556-supplementary.pdf]

Suppl. Material

Variables Recorded at Admission

- History and demographics: name, sex, age, newly diagnosed heart failure, medication, and comorbidities.
- Clinical examination: pulmonary crackles, edema graded 0 to +3, jugular venous distension at 45 degrees or hepatojugular reflux, and NYHA class.
- Laboratory testing: complete blood count, biochemistry including urea, creatinine, and sodium, admission NT-proBNP, admission and 2-hour spot urinary sodium, chloride, and creatinine, and blood gas analysis.
- Admission echocardiography: LVEF, left ventricular filling pressures by lateral E/e′, valvular disease severity, left atrial dimensions, right ventricular end-diastolic diameter, right ventricular systolic function by tissue Doppler S′, right ventricular systolic pressure, and inferior vena cava size and respiratory variation.
- Chest radiography: congestion or pulmonary edema and pleural effusion.
- Electrocardiography: rhythm, conduction abnormalities, and ischemic changes.
